# Mechanism and clinical role of TIMP-2 and IGFBP-7 in cardiac surgery-associated acute kidney injury: A review

**DOI:** 10.1097/MD.0000000000038124

**Published:** 2024-05-24

**Authors:** Jiajie Leng, Letai Li, Hongwen Tu, Yuxiang Luo, Zhenrui Cao, Kun Zhou, Syed M Musa Rizvi, Hongtao Tie, Yingjiu Jiang

**Affiliations:** a Department of Cardiothoracic Surgery, The First Affiliated Hospital of Chongqing Medical University, Chongqing, China; b Department of anesthesiology, The First College of Clinical Medicine, Chongqing Medical University, Chongqing, China.

**Keywords:** acute kidney injury, biomarkers, cardiac surgery, insulin-like growth factor-binding protein-7, tissue inhibitor of metalloproteinase-2

## Abstract

Acute kidney injury (AKI) is a common postoperative complication, but there is still a lack of accurate biomarkers. Cardiac surgery-associated AKI is the most common cause of major-surgery-related AKI, and patients requiring renal replacement therapy have high mortality rates. Early diagnosis, intervention, and management are crucial for improving patient prognosis. However, diagnosing AKI based solely on changes in serum creatinine level and urine output is insufficient, as these changes often lag behind actual kidney damage, making early detection challenging. Biomarkers such as tissue inhibitor of metalloproteinases-2 (TIMP-2) and insulin-like growth factor-binding protein-7 (IGFBP-7) have been found to be significant predictors of moderate-to-severe AKI when combined with urine content analysis. This article reviews the mechanism of biomarkers TIMP-2 and IGFBP-7 in AKI and provides a comprehensive overview of the clinical effects of TIMP-2 and IGFBP-7 in cardiac surgery-associated AKI, including prediction, diagnosis, and progression.

## 1. Introduction

Acute kidney injury (AKI), a common complication in intensive care, is characterized by a decline in glomerular filtration function and is associated with multiple etiologies and pathophysiological processes. It predominantly occurs in undergoing major surgeries and has high morbidity and mortality rates.^[1,2]^ Recent studies have revealed that, despite advancements in surgical techniques and postoperative management, the incidence of AKI remains between 30% and 59%, with severe cases having a mortality rate as high as 50.0%. Once AKI develops, it significantly increases the risk of postoperative infection and cardiovascular events for several years after surgery. Numerous studies have demonstrated that postoperative AKI leads to increased morbidity and mortality rates, elevated hospitalization costs, prolonged hospital stays, and can even progress to chronic or end-stage renal disease.^[3,4]^ Based on its etiology, AKI can be categorized as sepsis-related AKI, major-surgery-related AKI, renal hypoperfusion AKI, or nephrotoxic drug-induced AKI. Among these categories, cardiac surgery-associated AKI (CSA-AKI) is the most prevalent form of major-surgery-related AKIs and patients requiring renal replacement therapy (RRT) face an alarming mortality rate of up to 60%.^[5,6]^ Therefore, early diagnosis and timely intervention play a crucial role in improving patient outcomes after cardiac surgery.

Currently, the diagnosis of AKI primarily relies on changes in serum creatinine levels and urine output, both of which serve as indicators of renal function rather than direct evidence of renal injury or stress and are susceptible to various influencing factors (including age, sex, and metabolic status). Consequently, diagnosis of postoperative AKI is often delayed. Therefore, existing diagnostic methods face challenges for the early detection of AKI.^[7,8]^ Novel biomarkers for diagnosing AKI have demonstrated superior sensitivity and specificity compared to serum creatinine and urine output. These biomarkers include interleukin-18 (IL-18), neutrophil gelatinase-associated lipocalin (NGAL), liver-type fatty acid-binding protein (L-FABP), kidney injury molecule-1 (KIM-1), tissue inhibitor of metalloproteinase-2 (TIMP-2), urinary chitinase 3-like protein, human insulin-like growth factor-binding protein-7 (IGFBP-7), dickkopf-3, and cystatin C.^[9]^ In detail, IL-18, a 18 kDa pro-inflammatory cytokine, is released into urine following tubular damage with clinical function of diagnosing and predicting AKI.^[10,11]^ NGAL occur in urine and plasma at least 3 different types, including monomeric 25 kDa glycoprotein, homodimeric 45 kDa protein and heterodimeric 135 kDa protein. It is produced by tubular cells and neutrophils to diagnose the severity of AKI.^[12,13]^ L-FABP, a 14 kDa intracellular lipid chaperone produced by proximal tubule help diagnose AKI.^[14]^ KIM-1, a type of transmembrane glycoprotein released into urine, which predicted and identify the severity of AKI.^[15]^ Dickkopf-3 is secreted into urine under intensive stress of AKI, which help assess the risk of AKI.^[16]^ Cystatin C, 13 kDa cysteine protease inhibitor produced by nucleated human cells, is filtered into plasma and regarded as the biomarkers of diagnose and severity of AKI.^[17]^

Among these, IGFBP-7 and TIMP-2 exhibit functional diversity, encompassing the prediction, diagnosis, and severity assessment of AKI. These biomarkers actively participate in the initial stages of renal cell injury (RCI). Furthermore, IGFBP-7 and TIMP-2 are closely associated with AKI induced by factors, such as cardiac surgery, kidney transplantation, decompensated heart failure, cardiac arrest, sepsis, and toxic nephropathy. This study also demonstrated that the combination of urinary TIMP-2 and IGFBP-7 can be utilized to evaluate the risk of AKI in critically ill postoperative patients. In 2014, “NephroCheck” was approved by the U.S. Food and Drug Administration for the detection of TIMP-2 and IGFBP-7 levels in urine to predict the likelihood of AKI in critically ill patients. Dusse et al^[18]^ discovered that urinary TIMP-2 and IGFBP-7 possess prospective diagnostic potential, with an estimated area under the curve (AUC = 0.97) during the early stages of CSA-AKI. This article introduces the biological characteristics and functions of TIMP-2 and IGFBP-7, while reviewing their mechanisms of action and clinical roles in CSA-AKI. This further elucidates the clinical application of TIMP-2 and IGFBP-7 in early diagnosis following cardiac surgery to provide novel insights for relevant departments.^[19,20]^

## 2. The mechanism of protein in urine and gene regulation of TIMP-2 and IGFBP-7 in CSA-AKI

TIMP-2 protein, with a molecular weight of 22 kDa and solubility, is believed to be associated with leukocyte infiltration, cellular injury, and disruption of cell junctions. IGFBP-7, which has a molecular weight of 26 kDa and is soluble, is expressed in the kidney and other tissues and may be related to cell damage or induced by this process. Both biomarkers are secreted in the proximal convoluted tubule (PCT) and distal convoluted tubule (DCT). TIMP-2 is predominantly expressed in cells derived from DCT, whereas IGFBP-7 is preferentially secreted by cells derived from PCT. In cases of AKI, both biomarkers increase in urine due to kidney stress or injury and jointly participate in the early G1 phase cell cycle arrest processes.^[21,22]^ Schanz et al^[23]^ demonstrated that in human kidney tissue sections, increased mRNA and protein expression levels can be observed in renal disease associated with AKI. This is recognized as the mechanism of increased urinary TIMP-2 and IGFBP-7 proteins. Interestingly, Johnson and Zager^[24]^ proposed that the levels of the urinary proteins TIMP-2 and IGFBP-7 remained elevated, displaying a proportional increase with total urinary protein concentration in the absence of gene expression activation of TIMP-2 and IGFBP-7, thereby potentially indicating tubular cell leakage. Overall, the increased protein levels of TIMP-2 and IGFBP-7 in the urine seemed to be caused by multiple factors. The increased gene and protein expression of TIMP-2 and IGFBP-7, together with protein leakage in the AKI state, led to an observed increase in urinary content.

**Figure 1.**
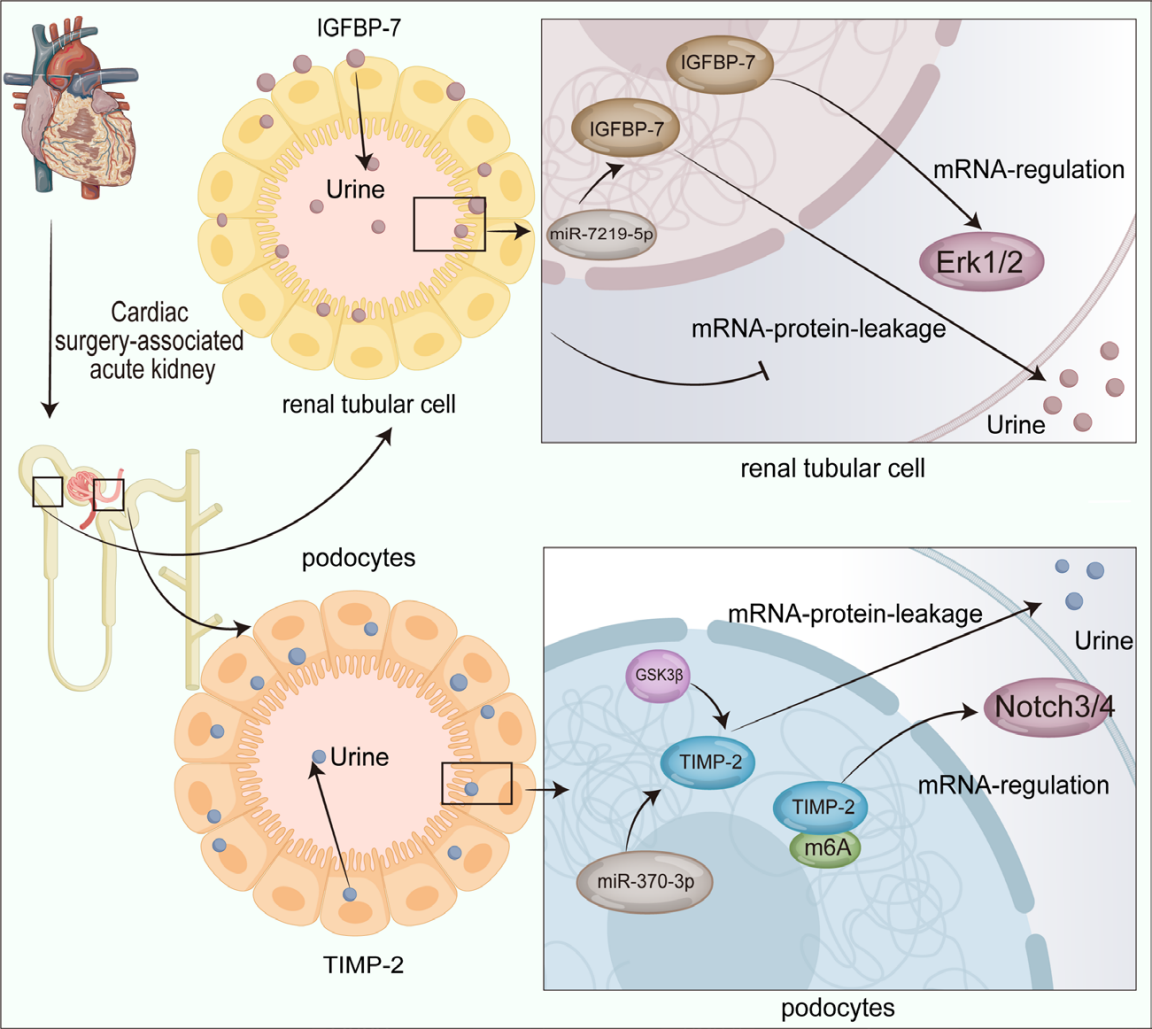
The mechanism of protein in urine and gene regulation of TIMP-2 and IGFBP-7 in CSA-AKI. CSA-AKI = cardiac surgery-associated AKI, IGFBP-7 = insulin-like growth factor-binding protein-7, TIMP-2 = tissue inhibitor of metalloproteinases-2.

The regulation of TIMP-2 and IGFBP-7 in AKI has been investigated in recent studies. Jiang et al^[25]^ demonstrated that the elevated m6A modification level of TIMP-2 exerts a protective effect on injured podocytes in mice with diabetic nephropathy, thereby attenuating inflammation and apoptosis of renal podocyte injury through the Notch3/4 signaling pathway. Guo et al^[26]^ suggested that the gene expression level of TIMP-2 is inhibited by GSK3β to eliminate the accumulation of extracellular matrix and attenuate acute podocyte injury in glomerulosclerosis. In addition, in septic AKI, TIMP-2 is recognized as the target of miR-370-3p, which is regulated by circ_0114428.^[27]^ TIMP-2-related therapeutic targets and mechanisms of regulation should be further explored in AKI. Meanwhile, the expression levels of IGFBP-7 can be inhibited by gypenoside XLIX to reduce programmed cell death and alleviate cisplatin-induced AKI.^[28]^ Wang et al^[29]^ demonstrated that the overexpression of IGFBP-7 significantly induced cell cycle arrest at the G1-G0 phases and promoted apoptosis in LPS-induced HK-2 cells. Knockdown of IGFBP-7 effectively ameliorated the severity of renal injury in sepsis-induced AKI mice, as evidenced by reductions in urinary levels of creatinine, blood urea nitrogen, and albumin and attenuation of cell apoptosis through ERK1/2 signaling activation. Additionally, IGFBP-7 can be activated by Circ_35953/miR-7219-5p/HOOK3 regulation and participates in the occurrence and development of sepsis-AKI.^[30]^ The current regulatory mechanism depicted in Figure 1 encompasses the proteinuria pathway and gene regulation of TIMP-2 and IGFBP-7 in CSA-AKI.

## 3. The clinical role of TIMP-2 and IGFBP-7 in CSA-AKI

### 3.1. TIMP-2 and IGFBP-7 for the prediction in CSA-AKI

In recent years, a substantial amount of research has been conducted on the predictive capabilities of TIMP-2 and IGFP7 in CSA-AKI, as evidenced by their inclusion in Table 1.^[31–45]^

**Table 1:** The research of TIMP-2 and IGFBP-7 in cardiac surgery-associated AKI.

Year	Author	AKI diagnostic criteria	AKI threshold	AKI/ALL	detection time	AUC	Cutoff	Sensitivity	Specificity
2014	Meersch et al^[31]^	KDIGO	AKI stage ≥ 1 within 72 h after surgery	26/50	4 h after CPB	0.81	0.3	0.8	0.83
				26/50	MVT	0.9	0.5	0.92	0.81
2014	Meersch et al^[32]^	RIFLE	CCl decrease of 50% within 72 h after surgery	12/50	4 h after CPB	0.85	0.7	0.83	0.77
2015	Pilarczyk et al^[33]^	KDIGO	AKI stage ≥ 2 within 48 h after surgery	19/60	4 h after CPB	0.861	0.15	0.83	0.67
					24 h after CPB	0.869	0.89	0.8	0.81
					Maximum value time	0.838	0.89	0.83	0.78
2015	Wetz et al^[34]^	KDIGO	AKI stage ≥ 1 within 24 h after surgery	16/42	24 h after CPB	0.706	0.3	0.53	0.54
2016	Dusse et al^[35]^	KDIGO	AKI stage ≥ 2 within 48 h after surgery	15/40	24 h after CPB	0.97	1.03	1	0.9
2017	Finge et al^[36]^	KDIGO	AKI stage ≥ 1 within 24 h after surgery	34/93	3 h after CPB	0.73	0.3	0.76	0.64
2017	Mayer et al^[37]^	KDIGO	AKI stage ≥ 1 within 24 h after Surgery	9/110	1 h after starting CPB	NP	0.4	0.78	0.64
2017	Oezkur et al^[38]^	KDIGO	AKI stage ≥ 1 within 48 h after surgery	35/150	At postoperative ICU admission	0.81	0.3	0.6	0.88
2017	Wang et al^[39]^	KDIGO	AKI stage ≥ 1 within 7 d after surgery	20/57	4 h after postoperative ICU admission	0.8	0.3	0.75	0.7
2018	Zaoute et al^[40]^	KDIGO	AKI stage ≥ 1 within 7 d after surgery	37/50	12 h after surgery	0.69	0.3	0.65	0.62
2020	Grieshaber et al^[41]^	KDIGO	AKI stage ≥ 2 within 6 d after surgery	133/613	24 h after CPB	0.63	0.3	0.38	0.81
2021	Couturier et al^[42]^	KDIGO	AKI stage ≥ 1 within 48 h after surgery	114/185	24 h after surgery	0.845	0.3	0.808	0.553
2021	Ramírez et al^[43]^	AKINI	AKINI stage ≥ 1 within 96 h after surgery	19/36	2 h after CBP	0.848	0.16	0.684	0.883
2022	Irqsusi et al^[44]^	KDIGO	AKI stage ≥ 1 within 48 h after surgery	14/50	0 h after surgery	0.725	0.07	0.846	0.556
					24 h after surgery	0.718	0.35	0.538	0.882
2023	Lacquaniti et al^[45]^	KDIGO	AKI stage ≥ 1 within 24 h after surgery	113/230	4 h after ICU	0.78	2	0.839	0.738

AKI = acute kidney injury, AUC = area under the receiver operating characteristic curve, CPB = cardiopulmonary bypass, DGF = delayed graft, function, ICU = intensive care unit, IGFBP-7 = insulin-like growth factor-binding protein-7, KDIGO = kidney disease improving global outcomes, MVT = Maximum TIMP-2*IGFBP-7 value time, TIMP-2 = time tissue inhibitor ofmetalloproteinases-2.

TIMP-2 and IGFBP-7 alone represent widely accepted and effective approaches for predicting AKI. After cardiac surgery in children with AKI, common and serious complications arise because of the main mechanism of nonpulsatile blood perfusion and systemic inflammatory reactions during extracorporeal circulation, leading to renal vasoconstriction, small arterial embolism, and subsequent renal ischemia–hypoperfusion. These complications can result in a poor prognosis in children. Therefore, it is crucial to eliminate these risk factors to prevent AKI. The early diagnosis and treatment of AKI can significantly improve the prognosis of children. Krawczeski et al^[46]^ conducted a study on 220 children after cardiac surgery and discovered that a postoperative 4-hour urine TIMP-2 × IGFBP-7 > 0.7 (ng/mL) had an AUC value of 0.85 for diagnosing AKI. TIMP-2 with IGFBP-7, as well as other biomarkers, can enhance the accuracy of predicting pre-AKI conditions. Overall, these biomarkers are more sensitive than creatinine; however, further research is needed to fully understand their use and significance in diagnosing AKI in children undergoing cardiac surgery for congenital heart disease.^[47,48]^ For adult with CSA-AKI, both postoperative TIMP-2 and postoperative IGFBP-7 are considered to be the key mechanism of AKI, Jia et al^[49]^ conducted a stent placement and angioplasty protection study in high-risk endarterectomy patients to identify and validate New biomarkers for AKI, the results showed that TIMP-2 and IGFBP-7 predicted the development of AKI within 12h with an AUC of 0.8. Some previous studies have shown that the urinary TIMP-2 × IGFBP-7 level in AKI patients related to cardiac surgery is significantly higher than that of non-AKI patients at 3 hours after surgery, which has diagnostic significance at 24 hours after surgery. The patient’s serum creatinine level changed significantly only 24 hours after surgery.^[50]^ Further statistical analysis showed that the 3-hour urine TIMP-2 × IGFBP-7 of cardiac surgery patients had good specificity and sensitivity for distinguishing AKI patients from non-AKI patients, and the 24-hour urine TIMP-2 × IGFBP-7 had better specificity. This suggests that urine TIMP-2 × IGFBP-7 levels at 3 hours after cardiac surgery could be used as a better early predictor of AK in cardiac surgery. In summary, this review suggests that early warning of CSA-AKI can be achieved by 3h postoperative urine TIMP-2 × IGFBP-7 levels, which can be used for early diagnosis and management. TIMP-2 × IGFBP-7 continued to increase, indicating that the patient’s diagnosis of AKI was correct, and active and effective intervention should be performed immediately to improve prognosis.^[51]^

In another cohort of 150 children with AKI following cardiac surgery, the biomarkers showed an increasing order: urine NGAL (2 hours after surgery) > urine IL-18 and urine L-FABP, urine TIMP-2, and IGFBP-7 (6 hours after surgery) > urine KIM-1 (12 hours postoperatively). These biomarkers were significantly elevated at earlier time points than serum creatinine levels at the onset of AKI. Among these markers, urinary NGAL exhibited the highest predictive power at each time point (AUC > 0.9). However, when combined with urinary IL-18 and TIMP-2 at 2 and 6 hours after pediatric cardiac surgery, the predictive power improved from an AUC of 0.938 to 0.973.^[52]^ This indicates that early stage biomarkers can predict AKI following major cardiac surgery, and utilizing multiple markers in combination can predict AKI. The study also evaluated the predictive capabilities of renal injury biomarkers in patients with postoperative AKI after major cardiac surgery, revealing that urinary TIMP-2 and IGFBP-7 markers effectively predicted AKI (AUC between 0.7 and 0.85). Another prospective multicenter study validated the aforementioned findings. The study included 375 patients across 39 surgical intensive care units in Europe and North America, with 35 patients (9%) developing moderate-to-severe disease within 12 hours post-surgery. Urinary TIMP-2, and IGFBP-7 demonstrate an AUC of 0.84 for predicting moderate-to-severe AKI, significantly enhancing the predictive performance of the clinical risk prediction model.^[53]^ Furthermore, Yan et al^[54]^ indicated that combining TIMP-2 and IGFBP-7 with clinical findings improved the predictive value of AKI compared to biomarkers alone; the combined model’s AUC (0.86) surpassed that of the individual predictive model. Postoperative NGAL, postoperative TIMP-2, postoperative IGFBP-7, and postoperative pCr prediction models based on the random forest algorithm can be implemented to forecast AKI incidence in patients undergoing cardiothoracic surgery. Studies have confirmed that TIMP-2 and IGFBP-7 serve as markers of renal tubular epithelial injury. When sepsis or ischemic injury occurs, they promptly transmit corresponding signals, causing renal tubular cells to arrest in the G1 cell cycle phase momentarily after entry, thus preventing further damage progression.^[55]^ These physiological and pathological mechanisms, along with cellular pathways, elucidate the potential application prospects of TIMP-2 and IGFBP-7 in early AKI prediction.

### 3.2. TIMP-2 and IGFBP-7 for the diagnosis in CSA-AKI

In view of the predictive ability of TIMP-2 and IGFBP-7 in AKI that has been found, their combination with the conventional indicators of creatinine and urine volume was attempted to enhance the diagnostic accuracy of AKI.^[56]^ The current clinical diagnostic criteria for AKI was formulated by the Global Kidney Disease Improvement Working Group in 2012, and the serum creatinine level should be increased by at least 0.3 mg dL^−1^ or more than 1.5 times the baseline within 48 hours (known or It is presumed to occur within the first 7 days) or the urine output drops below 0. 5 mL (kg h)^−1^ for 6 hours to define AKI stage 1.^[57,58]^ The modification of KDIGO stage 1 AKI proposed by Marlies Ostermann et al introduced 3 substages (namely, 1S, 1A, and 1 B) and further categorized stages 2 and 3 AKI based on the presence of biomarkers in the JAMA Network Open. They proposed stage 1S. The 3 substages of stage 1 are as follows: stage 1S was positive for novel biomarkers but negative for creatinine and urine volume. Stage 1A refers to being negative for novel biomarkers but positive for creatinine and urine volume. Stage 1B refers to being positive for both criteria.^[59]^ However, the proposal of these substages is solely supported by studies focusing on the NGAL biomarker.^[60,61]^ Further prospective trials are required to establish a similar definition of CSA-AKI for TIMP-2 and IGFBP-7 expression.

Additionally, TIMP-2 and IGFBP-7 demonstrated independent diagnostic performance. Meersch et al^[62]^ implemented interventions such as the KDIGO bundle on post-cardiac surgery patients to investigate the independent diagnostic capability of this biomarker, urinary TIMP-2 × IGFBP-7, with a threshold value > 0.3 (ng/mL)^2^/1000. The researchers attempted to utilize innovative biomarkers for diagnosis and corresponding interventions targeting AKI, resulting in significant reductions in the incidence of AKI within the intervention group (*P* = .004), as well as moderate-to-severe cases of AKI (*P* = .009).

The elevation of TIMP-2 and IGFBP-7 has a distinctive diagnostic potential in identifying progression from AKI stage 1. The analysis of long-term outcomes revealed that the risks associated with TIMP-2 × IGFBP-7 levels exceeding 2.0 (ng/mL)^2^/1000 were comparable to the progression of AKI, even in cases where no progression from AKI stage 1 was observed based on serum creatinine (sCr) and urine output. Currently, there is a lack of relevant studies to substantiate the superior diagnostic efficacy of TIMP-2 and IGFBP-7 compared to traditional markers, such as creatinine and urine volume. To advance our understanding, future investigations should focus on conducting cohort studies that explore combined diagnosis using both new and traditional markers, as well as independent diagnostic capabilities utilizing new markers.

### 3.3. TIMP-2 and IGFBP-7 for progression in CSA-AKI

The condition of patients with CSA-AKI will improve after treatment and management, potentially transitioning from persistent AKI to acute kidney disease (AKD) and chronic kidney disease (CKD).^[63]^ Therefore, Monitoring patient progression using biomarkers is crucial for prognosis assessment by clinical staff. Koyner et al conducted a large, international, prospective observational study. The urinary levels of TIMP-2 × IGFBP-7 were measured in patients with moderate-to-severe AKI following cardiac surgery, and their association with long-term outcomes (death or dialysis) at 9 months was compared. The levels of TIMP-2 × IGFBP-7 were categorized into 3 grades: >2.0, 0.3–2.0, ≤0.3, and demonstrated a significant association with unfavorable long-term consequences.^[64]^ Meersch et al^[31]^ investigated the predictive value of urine TIMP-2 × IGFBP-7 (AUC = 0.79) for RRT before discharge from AKI. A study conducted by Dusse et al^[35]^ demonstrated that the combination of maximum TIMP-2 × IGFBP-7 levels exhibits robust diagnostic efficacy for RRT, with an area under the curve (AUC) value of 0.919. Esmeijer et al^[65]^ conducted a study investigating the correlation between TIMP-2 **×** IGFBP-7 levels and the requirement for RRT following cardiac surgery; the preoperative and postoperative changes in urinary IGBP7 × TIMP-2 levels provided a reliable prediction of the need for RRT, while the postoperative urinary IGBP7 × TIMP-2 level served as a valuable indicator of 30-day mortality. In conclusion, TIMP-2 × IGFBP-7 is considered a more reliable indicator for predicting or diagnosing poor prognosis or even mortality in patients with acute renal failure following cardiac surgery. Early initiation of RRT is recommended to achieve favorable outcomes in patients with AKI.

## 4. Conclusion and prospect

AKI remains the primary complication of cardiac surgery and is attributed to the cumulative effects of multiple factors. The optimal prevention and treatment plan still lacks clarity, with emphasis on preventive measures and clinical strategies being the predominant approaches for effectively managing high-risk patients with AKI. Adopting a multi-model approach that considers risk factors associated with CSA-AKI is essential. Key aspects include evaluation, optimization of surgical techniques and procedures, and perioperative care to ensure comprehensive cardiovascular function management. Additionally, it is imperative to utilize advanced detection technologies to develop highly specific and sensitive biomarkers while reinforcing research efforts towards validating diagnostic tools. These endeavors aim to establish technical guidelines to prevent CSA-AKI development and facilitate renal function recovery.^[66–68]^

This review provides a comprehensive summary of the molecular mechanisms and clinical implications of the combination of TIMP-2 and IGFBP-7 in postoperative AKI. Notably, TIMP-2 combined with IGFBP-7 demonstrated robust predictive capability for short-term onset and long-term prognosis, thus exhibiting promising clinical potential. The early prediction and diagnosis of CSA-AKI are crucial for guiding timely clinical intervention and management, thereby enhancing patient prognosis. The early emergence of TIMP-2 and IGFBP-7, along with their noninvasive nature, high specificity, independence from race and gender influences, and easy specimen accessibility, have garnered widespread recognition for their detection. Furthermore, the combined detection of these biomarkers has shown great potential for improving the accuracy of prediction and diagnosis.^[69]^ The clinical significance of TIMP-2 and IGFBP-7 in predicting CSA-AKI is substantial. These biomarkers could serve as early indicators for monitoring AKI development in this patient population. Moreover, the convenience and rapidity of this detection method make it highly applicable for clinical use in monitoring renal injury in post-cardiac surgery patients.^[70,71]^ However, its diagnostic efficacy remains unclear. Further verification is necessary to independently evaluate its diagnostic efficacy as well as its combination with other biomarkers. In the future, a research direction is to identify a combination of simple detection methods with low cost and high accuracy for joint biomarker detection.^[72]^ In summary, the research on relevant biomarkers for the prediction and diagnosis of CSA-AKI, on the one hand, is to seek stability and sensitivity, and on the other hand, the specific biomarkers should continue to explore the accuracy and clinical applicability.

## Author contributions

**Investigation:** Jiajie Leng.

**Methodology:** Jiajie Leng.

**Project administration:** Jiajie Leng.

**Visualization:** Jiajie Leng, Hongwen Tu, Zhenrui Cao, Kun Zhou, Syed M. Musa Rizvi, Hongtao Tie, Yingjiu Jiang.

**Writing – original draft:** Jiajie Leng, Letai Li, Hongtao Tie.

**Supervision:** Hongwen Tu, Yuxiang Luo, Zhenrui Cao, Syed M. Musa Rizvi.

**Validation:** Hongwen Tu, Zhenrui Cao, Kun Zhou, Syed M. Musa Rizvi, Hongtao Tie, Yingjiu Jiang.

**Conceptualization:** Kun Zhou.

**Software:** Syed M. Musa Rizvi.

**Writing – review & editing:** Hongtao Tie, Yingjiu Jiang.
